# Experimental Verification of a Coordinated Path-Following Strategy for Underactuated Marine Vehicles

**DOI:** 10.3389/frobt.2019.00035

**Published:** 2019-05-07

**Authors:** Dennis J. W. Belleter, José Braga, Kristin Y. Pettersen

**Affiliations:** ^1^Department of Engineering Cybernetics, Centre for Autonomous Marine Operations and Systems (NTNU AMOS), Norwegian University of Science and Technology, Trondheim, Norway; ^2^OceanScan Marine Systems and Technology, Lda, Matosinhos, Portugal

**Keywords:** underwater robotics, marine systems, sea-trials, multi-agent systems, coordinated path following

## Abstract

This work presents the results of an experimental verification of a coordinated path following strategy for underactuated marine vehicles. The coordinated path following strategy is presented, and is then experimentally verified using three autonomous underwater vehicles. The vehicles are required to coordinate their motion along spatially separated straight-line paths to obtain a desired formation. The vehicles are steered to the paths using an integral line-of-sight guidance approach that allows the vehicles to reject constant ocean currents. Simultaneously, the coordination is achieved by adjusting the velocity based on the along-path distance. First, simulation results are presented, which serve as benchmarks for the experimental results. Furthermore, the simulations are used to show the effect of changing different parameters. The simulation results are performed using high-fidelity hardware simulation models. The results obtained from experiments in the harbor of Porto are then presented and compared with the results of the simulation.

## 1. Introduction

Multi-vehicle systems have been the topic of a lot of research over the previous decades. This research has often been limited to the theoretical level such as consensus protocols based on graph theoretical considerations, coverage control algorithms or formation/coordinated path following. The works considering experimental verification of this theory on actual robotic systems have been much more limited. Furthermore, both theoretical results and experimental verification have been very limited for multi-agent systems consisting of marine vehicles where the agents are underactuated.

Within the multi-vehicle systems research community, one of the fields that has received a lot of interest is formation control. Comprehensive reviews of the literature in this field are given in Chen and Wang (2005) and Oh et al. (2015). Formation control strategies aim to drive agents to adhere to prescribed constraints on their states. These constraints typically include a desired relative inter-agent distance such that the vehicles achieve a formation. Different methods of achieving the formation goal have been investigated. The choice between the different methods is often motivated by the constraints of the vehicles, their inter-agent communication capacities, and the intended application for the formation. Three major research directions within the formation control field are leader-follower synchronization, the virtual structure approach, and coordinated path following.

Leader-follower synchronization is conceptually one of the simplest strategies for formation control. This is a hierarchical formation control approach in which there is a leader and there are followers. The leader can be allowed to move freely or has the task to follow a certain path or trajectory. However, the leader does not carry any responsibility for achieving the formation. This responsibility falls on the followers, which have the task to control their inter-agent position and/or orientation with respect to the leader to a desired value. The advantage of the leader-follower structure is that only local information needs to be used to achieve the formation. The disadvantages is that there is limited fault tolerance. In particular, if the leader fails the entire formation breaks down, and if one or more of the followers fail the leader does not change its behavior accordingly and the formation breaks down. A special form of leader-follower formation control is the one-to-one communication formation control, where each vehicle only receives information from one vehicle, its leader, and sends information to only one vehicle, its follower. Leader-follower synchronization for marine vessels is considered in Breivik et al. (2008), in which a leader-follower scheme for fully actuated marine vessels is presented that can be used both in a centralized and a decentralized control strategy. In the marine systems literature work on leader-follower synchronization has played an important part in research on underway replenishment of ships, see for instance Fu and Haddad (2003), Kyrkjebø et al. (2007), and Skejic et al. (2009). For these operations the supply-ship is usually responsible for synchronizing its motion with the ship it is supplying. In Kyrkjebø et al. (2007), the case of a fully actuated follower that synchronizes its output with a leader with unknown dynamics is investigated. An observer-controller scheme is utilized to achieve synchronization where the observers are used to estimate the unknown velocities of the leader and follower. The observer-controller scheme utilized in Kyrkjebø et al. (2007) is based on the theory for master-slave synchronization of robotic manipulators investigated in Nijmeijer and Rodriguez-Angeles (2003). In Skejic et al. (2009), the focus is on the interaction forces between two vessels during underway replenishment operations. For control purposes, the constant bearing guidance algorithm from Breivik and Fossen (2008) is used to synchronize the ships along a straight-line path. The vessels are underactuated, but no analysis of the underactuated internal dynamics are given. In Fu and Haddad (2003), underway replenishment between fully actuated vessels is investigated and adaptive backstepping controllers are designed to reject exogenous disturbances. In Peng et al. (2013), formation control of underactuated vessels under the influence of constant disturbances is considered using neural network adaptive dynamic surface control in a leader-follower scheme. In Wang et al. (2017), a leader-follower approach for unmanned surface vessels is considered. The vehicles track each other using vision sensors, and the work takes into account limited sensor ranges. The work, however, only considers vessels with diagonal mass and damping matrices and no environmental disturbances. The strategy is extended to autonomous underwater vehicles in Pang et al. (2018).

In the virtual structure approach, the goal for the individual vehicle is to converge to different points of a virtual structure. The virtual structure is usually a geometrically-rigid object that defines the shape of the formation. Consequently, when each vehicle is at its desired point on the virtual structure, the vehicles are in formation. Using this approach it is very straightforward to describe the desired overall behavior of the formation by appropriately designing the virtual structure and its motion. For marine systems, a virtual structure is used in the work of Skjetne et al. (2002). The approach uses a centralized control law to control the formation that generates inputs for the decentralized controllers of the vessels to achieve and maintain their position in the formation. This approach is decentralized and validated experimentally in Ihle et al. (2004). Another decentralized approach for marine surface vessels is developed in Ihle et al. (2006), in which the virtual structure is modeled as a set of mechanical constraints on the vehicles using Lagrangian mechanics. The reaction forces generated from violating these constraints are then used to control each vessel to keep the formation. Recent applications of the virtual structure method can be found in Zhang et al. (2016) and Yin et al. (2017). In Zhang et al. (2016), cooperative localization for vehicles using a virtual structure approach is investigated. Only the vehicle kinematics are considered and disturbances are not considered. In Yin et al. (2017), the virtual structure approach is considered for path following of curved paths. Vehicles are considered on a dynamic level, but environmental disturbances are not considered.

Coordinated path following is a two-fold approach to formation control. That is, a path is assigned to each vehicle individually. The vehicle is individually responsible for following this path. The formation is then achieved by coordinating the motion of the vehicles along their given paths. This allows for decentralized approaches in which only minimal information such as inter-agent distances need to be communicated. This type of formation control is best suited for formation control along straight lines and identical parallel curved paths, since when vehicles are on different curved paths it is non-trivial to find a suitable metric of inter-agent distance to synchronize the vehicles' positions. Therefore, this problem is mainly investigated in the marine systems literature since these types of paths are common trajectories in the marine systems literature. In Lapierre et al. (2004), path following of two underwater vehicles is investigated. The vehicles follow parallel paths to satisfy their path-following task, whilst achieving and maintaining a desired along-path distance to satisfy a coordination task. Similar results can be found in Børhaug et al. (2006) for surface vessels and Børhaug et al. (2007) for underwater vehicles. The work from Børhaug et al. (2006) is extended in Børhaug et al. (2011) to include a thorough study of the coordination dynamics using techniques from graph theory. The work in Lapierre et al. (2004) is a simplified version of this problem where one of the vehicles is responsible for coordinating the inter-agent distances along the path, which results in a leader-follower type coordinated path following. The work in Børhaug et al. (2011), is analyzed for a much wider range of communication topologies. The work in Ghabcheloo et al. (2006) and Ghabcheloo et al. (2009), considers coordinated path following in the presence of communication failures and time delays. In Ghabcheloo et al. (2006) and Ghabcheloo et al. (2009), the individual vehicles converge to a virtual target on the path to achieve path following. The motion of these virtual targets is then adjusted around their common nominal value based on their relative distances, to achieve coordination of the virtual targets and indirectly of the vehicles. In all the formation control approaches discussed above, the effects of ocean currents are not taken into account. Ocean currents are considered in, for instance, Almeida et al. (2010) and Ihle et al. (2007). However these works consider fully actuated marine vehicles. In Almeida et al. (2010), backstepping based controllers are derived for path following while coordination along the paths is performed using measurements of the inter-agent distances between vehicles. In Ihle et al. (2007), a path-following approach is used that is shown to satisfy passivity properties. This passive path-following strategy is combined with a coordination law that is also passive, which results in a passive closed-loop system. In Burger et al. (2009), line-of-sight (LOS) path-following with a conditional integrator is used for path following under the influence of unknown disturbances. However, the coordination dynamics are not analyzed in this work. Recent works considering coordinated path following are Wang et al. (2016) and Liu et al. (2016). In, Wang et al. (2016), coordinated path following is investigated theoretically for vehicles on curved paths with disturbances at a dynamic level. In Liu et al. (2016), coordinated path-following theory is investigated at a kinematic level and a state observer is used to estimate the necessary side-slip angle to compensate for disturbances.

The control strategy verified in this work falls into the category of coordinated path following and was proposed and analyzed in Belleter and Pettersen (2014). This strategy aims to steer the underactuated vehicles to a desired path individually, using a guidance law that can reject an unknown ocean current. The velocity commands of the vehicles are then used to coordinate the motion of the vehicles along their respective paths. In this work the coordinated path following strategy derived in Belleter and Pettersen (2014) is verified experimentally using three underactuated autonomous underwater vehicles. The vehicles are directed to paths consisting of concentric rectangles, on which their along-path distances are coordinated to achieve a line formation. The outline of this work is as follows. The guidance and coordination laws, which were proposed and analyzed in Belleter and Pettersen (2014), are presented in section 2. Section 3 presents the experimental setup. This is followed by results from a simulation benchmark and results from the experimental verification in section 4. In section 5, we present simulation results for three different cases, to show the influence of different parameters on the results, and use this to compare with and further interpret the experimental results. Finally, conclusions are presented in section 6.

## 2. The Guidance and Coordination Laws

This section presents the guidance principle that is used to steer the vehicles to the correct path and the coordination law that is used to coordinate the vehicle's along-path distance. A theoretical analysis of the combination of the integral line-of-sight guidance law and this coordination law for the model of an underactuated surface vessel can be found in Belleter and Pettersen (2014). The model of the vehicle is of the form:

(1a) $$\begin{array}{ll} \dot{\eta }= & R\left(\psi \right)\nu_{r}+V \end{array}$$

(1b) $$\begin{array}{ll} M{\dot{\nu }}_{r}+C\left(\nu_{r}\right)\nu_{r}+D\nu_{r}= & Bf \end{array}$$

where η ≜ [*x, y*, ψ]^*T*^ describes the position of the center of gravity and the orientation of the vehicle with respect to the inertial frame, $\nu_{r}≜{\left[u_{r},v_{r},r\right]}^{T}$ contains the surge, the sway and the yaw velocities, respectively, *M* is the mass matrix, *C*(ν_*r*_) is the Coriolis matrix, *D* is the damping matrix, *B* is the thrust allocation matrix, and *f* ≜ [*T*_*u*_, *T*_*r*_] is the vector of control inputs composed by the surge thrust and the rudder angle inputs *T*_*u*_ and *T*_*r*_, respectively.

For port-starboard symmetric vehicles, the matrices (*M, B, C, D*) have the following structure

(2a) $$\begin{array}{ll} M≜ & \left[\begin{array}{ccc} m_{11} & 0 & 0\\ 0 & m_{22} & m_{23}\\ 0 & m_{32} & m_{33} \end{array}\right];B≜\left[\begin{array}{cc} b_{11} & 0\\ 0 & b_{22}\\ 0 & b_{32} \end{array}\right]; \end{array}$$

(2b) $$\begin{array}{ll} C≜ & \left[\begin{array}{ccc} 0 & 0 & -m_{22}v_{r}-m_{23}r\\ 0 & 0 & m_{11}u_{r}\\ m_{22}v_{r}+m_{23}r & -m_{11}u_{r} & 0 \end{array}\right]; \end{array}$$

(2c) $$\begin{array}{ll} D≜ & \left[\begin{array}{ccc} d_{11} & 0 & 0\\ 0 & d_{22} & d_{23}\\ 0 & d_{32} & d_{33} \end{array}\right]. \end{array}$$

It is worth noting that the model (1) is valid for low speed motion, for which the damping can be assumed to be linear. Specifically, at low speed the non-linear damping effects can be neglected (Fossen, 2011, p.152–157). Furthermore, since *f* ∈ **R**^2^, the vehicle is under-actuated in the work space **R**^3^. This latter fact implies that the vehicle is not directly actuated in the sway direction, that is, sideways. Moreover, given the structure of the matrix *B* in (2a), it is easy to see that the control input in yaw *T*_*r*_, indirectly affects the sway direction. However, according to Fredriksen and Pettersen (2004), for port-starboard symmetric vehicles, there exists a point on the center line of the vehicle ahead of the center of gravity called the pivot point. In Fredriksen and Pettersen (2004), it is shown that when a change of coordinates to express the model (1) around the pivot point is applied. Then, the yaw control does not effect the sway motion. Hence, the dynamical model with the body-fixed frame positioned at the pivot point is the following:

(3a) $$\begin{array}{ll} \dot{x} & =u_{r}\cos\left(\psi \right)-v_{r}\sin\left(\psi \right)+V_{x} \end{array}$$

(3b) $$\begin{array}{ll} \dot{y} & =u_{r}\sin\left(\psi \right)+v_{r}\cos\left(\psi \right)+V_{y} \end{array}$$

(3c) $$\begin{array}{ll} \dot{\psi } & =r \end{array}$$

(3d) $$\begin{array}{ll} {\dot{u}}_{r} & =F_{u_{r}}\left(v_{r},r\right)-\frac{d_{11}}{m_{11}}u_{r}+\tau_{u} \end{array}$$

(3e) $$\begin{array}{ll} {\dot{v}}_{r} & =X\left(u_{r}\right)r+Y\left(u_{r}\right)v_{r} \end{array}$$

(3f) $$\begin{array}{ll} \dot{r} & =F_{r}\left(u_{r},v_{r},r\right)+\tau_{r}. \end{array}$$

where τ_*u*_ and τ_*r*_ are, respectively, the surge force and the yaw torque input. The functions *X*(*u*_*r*_), *Y*(*u*_*r*_), *F*_*u*_, and *F*_*r*_ are given by

(4a) $$\begin{array}{ll} F_{u_{r}}\left(v_{r},r\right) & ≜\frac{1}{m_{11}}\left(m_{22}v_{r}+m_{23}r\right)r \end{array}$$

(4b) $$\begin{array}{ll} X\left(u_{r}\right) & ≜\frac{m_{23}^{2}-m_{11}m_{33}}{m_{22}m_{33}-m_{23}^{2}}u_{r}+\frac{d_{33}m_{23}-d_{23}m_{33}}{m_{22}m_{33}-m_{23}^{2}} \end{array}$$

(4c) $$\begin{array}{ll} Y\left(u_{r}\right) & ≜\frac{\left(m_{22}-m_{11}\right)m_{23}}{m_{22}m_{33}-m_{23}^{2}}u_{r}-\frac{d_{22}m_{33}-d_{32}m_{23}}{m_{22}m_{33}-m_{23}^{2}} \end{array}$$

(4d) $$\begin{array}{l} \begin{array}{c} F_{r}\left(\cdot \right)≜\frac{m_{23}d_{22}-m_{22}\left(d_{32}+\left(m_{22}-m_{11}\right)u_{r}\right)}{m_{22}m_{33}-m_{23}^{2}}v_{r}\\ \;+\frac{m_{23}\left(d_{23}+m_{11}u_{r}\right)-m_{22}\left(d_{33}+m_{23}u_{r}\right)}{m_{22}m_{33}-m_{23}^{2}}r. \end{array} \end{array}$$

Note that the functions *X*(*u*_*r*_) and *Y*(*u*_*r*_) are linear functions of the velocity. The yaw controller τ_*r*_ of the model is a feedback linearizing PD controller:

(5) $$\begin{array}{l} \tau_{r}=-F_{r}\left(u_{r},v_{r},r\right)+{\ddot{\psi }}_{d}-k_{\psi }\left(\psi -\psi_{d}\right)-k_{r}\left(\dot{\psi }-{\dot{\psi }}_{d}\right), \end{array}$$

with *k*_ψ_ > 0 and *k*_*r*_ > 0 constant controller gains. This controller assures that ψ and *r* exponentially track ψ_*d*_ and ${\dot{\psi }}_{d}$, respectively. The guidance law to describe the desired yaw angle is presented in section 2.1. The speed controller τ_*u*_ is chosen as a feedback linearizing P controller:

(6) $$\begin{array}{l} \tau_{u}=-F_{u_{r}}\left(v_{r},r\right)+\frac{d_{11}}{m_{11}}u_{c}+{\dot{u}}_{c}-k_{u_{r}}\left(u_{r}-u_{c}\right), \end{array}$$

with *k*_*u*_*r*__ > 0 a constant controller gain. The choice for the desired speed to achieve coordination is presented in section 2.2.

The ocean current satisfies the following assumption.

Assumption 1. *The ocean current is assumed to be a constant in time, uniform in space, and irrotational with respect to the inertial frame, i.e.*, $V_{c}≜{\left[V_{x},V_{y},0\right]}^{T}$. *Furthermore, there exists a constant* *V*_max_ > 0 *such that* $\|V_{c}\|=\sqrt{V_{x}^{2}+V_{y}^{2}}\leq V_{\max}$.

This assumption for the disturbance covers slowly time-varying disturbances, such as ocean currents, for which it is a widely accepted practice to model them as a constant drift force Fossen (2011). Furthermore, it accounts for part of the disturbances caused by waves for which the effects can be separated into first-order and second-order effects Fossen (2011). The first-order wave-induced forces cause wave-frequency induced motion and are observed as zero-mean oscillatory motions. The second-order wave-induced forces are wave drift forces that are observed as nonzero slowly varying components. Hence, the second-order effects cause a drift force similar to the ocean current and also satisfy Assumption 1. The first-order wave-induced forces cause zero-mean oscillatory motions that should not be compensated for by the vehicles actuators since this would cause oscillations in the vehicle propulsion and rudder system, something which is undesirable since it leads to extra wear and tear and power consumption.

The guidance and coordination law developed for the model above is presented in the next subsections.

### 2.1. Guidance Law

**Figure 1:**
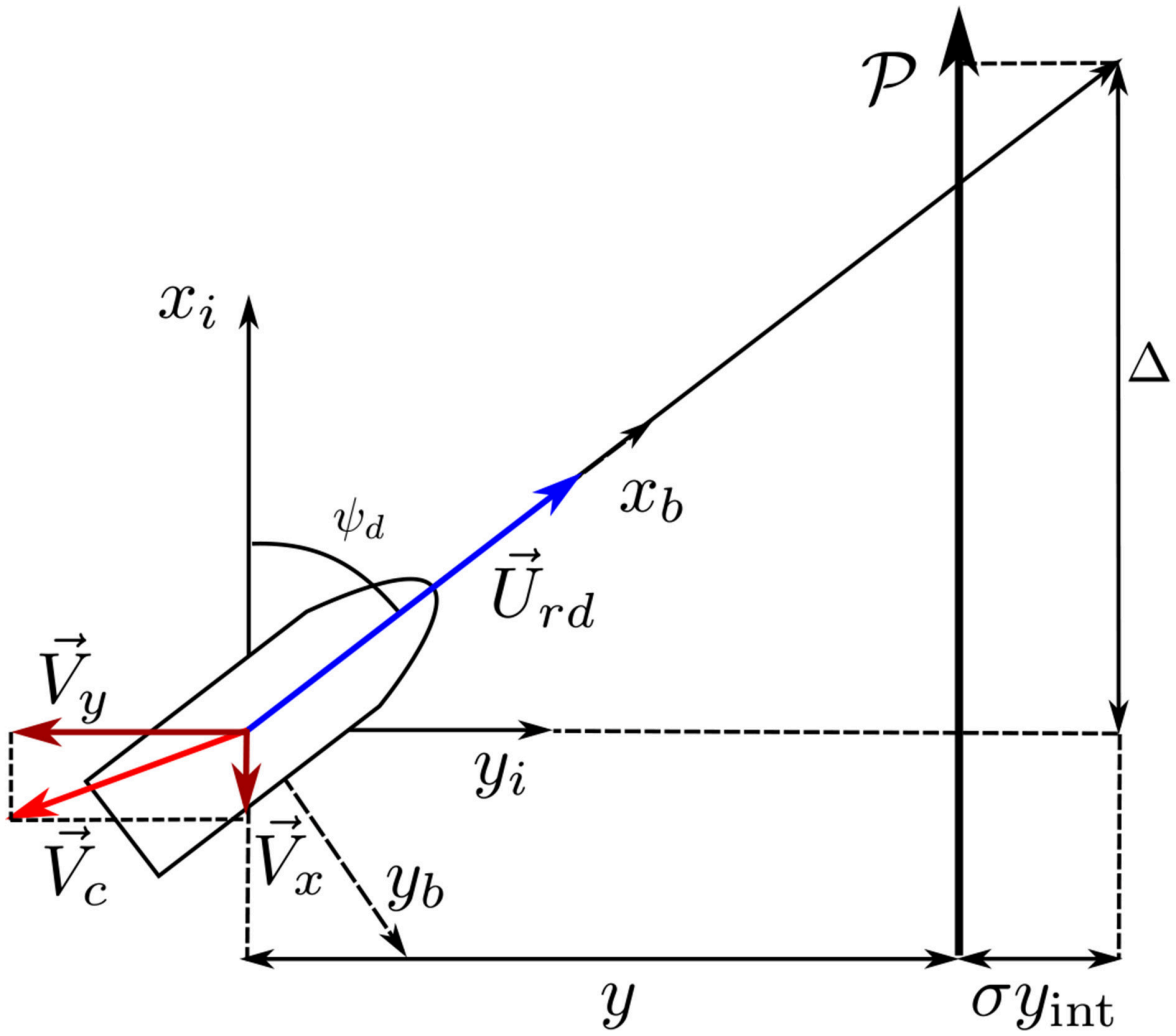
Illustration of the integral line-of-sight guidance.

The guidance law that is used is an integral line-of-sight guidance law. This guidance law was first introduced by Børhaug et al. (2008) and assigns the desired heading angle based on the cross-track error *y* − *D*_*j*_, an adaptive part to add the integral action that is used to compensate for the unknown ocean current, σ*y*_int_, and the look-ahead distance Δ, resulting in the desired heading angle assignment:

(7a) $$\begin{array}{ll} \psi_{d} & ≜-\tan^{-1}\left(\frac{\left(y-D_{j}\right)+\sigma y_{\text{int}}}{\Delta }\right),\;\Delta >0, \end{array}$$

(7b) $$\begin{array}{ll} {\dot{y}}_{\text{int}} & =\frac{\Delta \left(y-D_{j}\right)}{{\left(\left(y-D_{j}\right)+\sigma y_{\text{int}}\right)}^{2}+\Delta^{2}}, \end{array}$$

where σ is the integral gain, *y*_int_ is the state of the integrator, *y* is the distance to the path, and *D*_*j*_ is a desired offset of the path belonging to vehicle *j*. An illustration of the guidance law can be seen in Figure 1. The look-ahead distance Δ is chosen as a trade-off between convergence rate and stability. Specifically, a larger Δ would mean slower convergence to the path, while if Δ is chosen too small the vehicle will oscillate around the path. As shown in Børhaug et al. (2008), the integral action allows the yaw angle assignment (7a) to be non-zero when the vehicle is on the desired path, which in turn allows the vessel to compensate for the ocean current component perpendicular to the path. To reduce the risk of integrator wind-up, the integrator update law (7b) is defined such that the right hand side becomes small when the cross-track error is large, to avoid integral wind-up.

### 2.2. Coordination Law

The coordination objective is defined in terms of the along-path position of multiple vessels. The along-path position is a metric for the distance traversed by a vehicle along its straight-line path. If the vehicles have to make a corner, the corner section will have to be normalized to keep the path length identical for each vehicle. To achieve a desired formation, the vessels need to communicate their along-path position and adjust their respective speeds based on their along-path position. To describe the communication between the LAUVs we utilize graph theory (see for instance Mesbahi and Egerstedt, 2010).

The communication network is represented by a directed graph or digraph G(V,E), where *V* is a set of vertices and *E* a set of edges. The vertices represent the vessels in the formation and the number of vertices is equal to the number of vessels. The edges represent communication channels and are represented by pairs of vertices. More specifically, if there is information transfer from vertex *v*_*i*_ to *v*_*j*_ then the pair (*v*_*j*_, *v*_*i*_) ∈ *E*.

The neighborhood $A_{j}$ of *v*_*j*_ is the set of vertices *v*_*i*_ ∈ *V* such that there is an edge from *v*_*j*_ to *v*_*i*_. Hence, when controlling vessel *j* only the along-path position *x*_*i*_ of the vessels where $i\in A_{j}$ may be used. The above allows us to give some definitions, based on Godsil and Royle (2001), that are used in the analysis of the formation dynamics in Belleter and Pettersen (2014). A vertex *v*_*k*_ ∈ *V* reachable from vertex *v*_*i*_ ∈ *V* if there is a path from *v*_*i*_ to *v*_*k*_. A vertex is globally reachable if it can be reached, either directly or indirectly, from every vertex in G(V,E). The graph is said to be strongly connected, if all vertices of G(V,E) are globally reachable.

Assumption 2. *It is assumed the communication graph has at least one globally reachable vertex*.

The coordination in Belleter and Pettersen (2014) is achieved by a coordination law at the velocity level that uses the desired surge speed assignment

(8) $$\begin{array}{l} u_{c_{j}}=U_{rd}-g(\underset{i\in A_{j}}{\sum }\left(x_{j}-x_{i}-d_{ji}\right)), \end{array}$$

consisting of the desired constant relative surge velocity *U*_*rd*_ and a variable speed assignment *g*(·) which is a function of the difference between the along-path position of vehicle *i*, vehicle *j*, and the desired along-path distance between the vessels *d*_*ji*_. The function *g*(*x*):ℝ → ℝ should be a continuously differentiable saturation-like function that satisfies

(9) $$\begin{array}{l} \begin{array}{c} -a\leq g\left(x\right)\leq a,\;\forall x\in \mathbb{R},\;g\left(0\right)=0,\\ 0<g^{\prime }\left(x\right)\leq \mu ,\forall x\in \mathbb{R},\;g^{\prime }\left(x\right)≜dg/dx \end{array} \end{array}$$

where *a* is the magnitude maximally allowed deviation from the nominal velocity *U*_*rd*_, and μ > 0 is an arbitrary constant. This also implies that the function *g*(*x*) should be a sector function belonging to the sector [0, μ]. A suitable choice for *g*(*x*) that satisfies the requirements set above is

(10) $$\begin{array}{l} g(\underset{i\in A_{j}}{\sum }\left(x_{j}-x_{i}-d_{ji}\right))≜\frac{2a}{\pi }\tan^{-1}(\underset{i\in A_{j}}{\sum }\left(x_{j}-x_{i}-d_{ji}\right)). \end{array}$$

which is the type of function we will use in the experimental verification.

## 3. Experimental Set-Up and Mission Description

In this section we present the experimental set-up that was used in the experimental verification and we present the description of the mission that was conducted.

### 3.1. Vehicle Description and Capabilities

**Figure 2:**
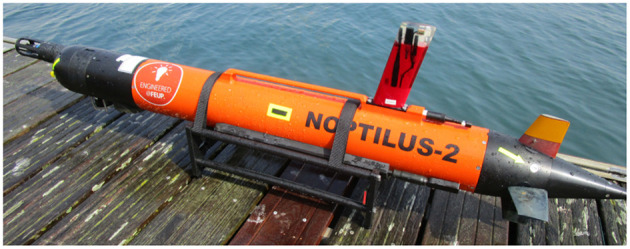
Image of one of the LAUVs used during the experiments.

The agents in the network were Ocean Scan Light Autonomous Underwater Vehicles (LAUVs). A picture of one of the LAUVs can be seen in Figure 2. The LAUVs are approximately 110 [cm] long depending on the configuration and have a diameter of 15 [cm]. The vehicles are designed to be light-weight and to be portable by one person. Therefore, their weight is between *15-20* [kg] depending on the configuration. The vehicles are rated for a maximum depth of 100 [m]. For propulsion the vehicle uses a DC motor coupled to a three-blade propeller. The propulsion system allows the LAUV to reach speeds of approximately 2 [m/s]. To steer the orientation, the LAUV is equipped with four fins. The vertically placed fins are used as rudders to control the yaw rotation. The horizontally placed fins are used as control surfaces to control the pitch rotation that is used to control the depth. To fit the model from (3), the depth controller is only used to assure that the vehicle stays at the surface. The vehicles are equipped with GPS so that they can get position measurements when at the surface. For underwater navigation the vehicles are equipped with acoustic modems, long baseline (LBL) navigation, ultra-short baseline (USBL) navigation, a Doppler velocity log (DVL), and a forward looking sonar for obstacle avoidance. For communication purposes the vehicles are equipped with an antenna that allows communication through WiFi and GSM/HSDPA.

### 3.2. Software Toolchain

The vehicles are operated using the open-source toolchain developed at the Underwater Systems and Technology Laboratory (LSTS) at the University of Porto. This toolchain consists, among others, of the unified navigation environment DUNE, the inter-module communication protocol (IMC), and Neptus which is the command and control software. DUNE is the on-board software running on the vehicles and communication gateways. The software is responsible for interactions with sensors, payload, and actuators and also takes care of anything related to communications, navigation, control, maneuvering, plan execution and vehicle supervision. To extend the functionality of the vehicles, source code can be added to the DUNE code repository. This is usually done as separate tasks that can interact with all other aspects on DUNE by exchanging data through the message bus system. For this communication, both on-board the vehicles and between vehicles, IMC is used. IMC consists of a set of messages common to all entities in the system, e.g., vehicles and communication nodes, and allows data exchange and communication. Hence, each entity of the network runs DUNE tasks to function, while IMC provides data-exchange and communication capabilities between vehicles and between different processes on the vehicles. The command and control software Neptus can be used during all different phases of a typical mission life cycle: planning, simulation, execution and post-mission analysis. It provides a graphical user interface with profiles of the available vehicles that include the sensory and maneuvering capabilities of vehicles. Moreover, it offers different types of geographical maps. All of this can be used to plan and simulate missions considering all aspects of the mission including battery life, available sensors, etc. During execution, Neptus can be used to visualize incoming real-time data from the operation, allows for tele-operation of vehicles, and can be used to send new maneuvering commands to the vehicles. In the review and analysis phase, Neptus can be used to process and visualize all the data stored through IMC messages of each vehicle. This allows the user to visualize and analyse the data.

### 3.3. Mission Description and Implementation

**Figure 3:**
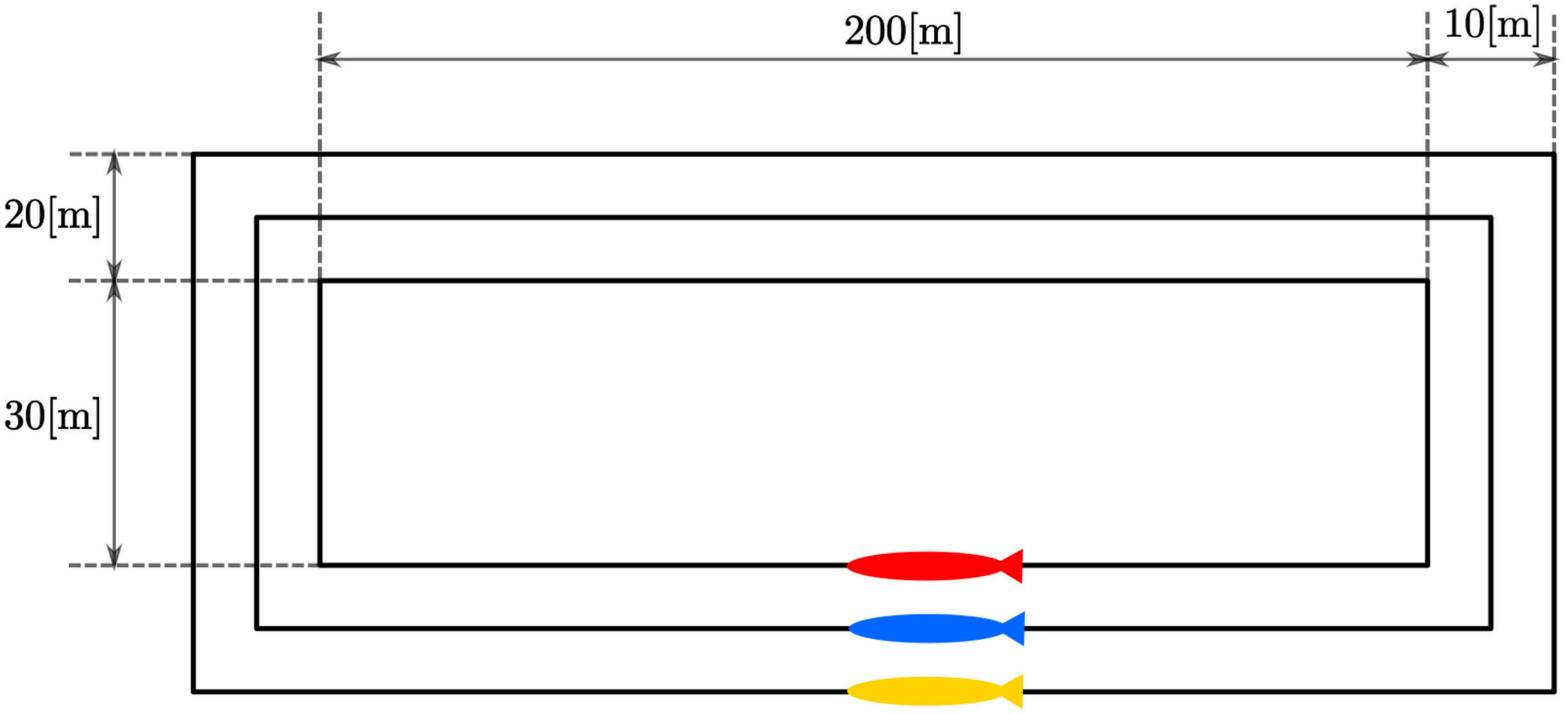
LAUV paths with dimensions.

The mission included the coordination of three LAUVs. The vehicles were required to coordinate their motion along parallel, spatially separated straight-line paths to obtain a desired formation. The experiment was performed in the harbor of Porto. To contain the motion of the LAUVs they were assigned rectangular-shaped paths. The motion pattern can be seen in Figure 3. From Figure 3 it can be seen that the length of the path was not the same for each LAUV. The paths were placed this way to avoid collisions between the LAUVs during cornering. However, this had the adverse effect that the path of the red LAUV on the inside was shorter than the path of the yellow LAUV on the outside trajectory. In fact, the length of the corner section for the yellow LAUV was 30 [m] longer than that of the blue LAUV and 60 [m] longer than that of the red LAUV. Consequently, the corner sections had to be normalized such that each vehicle's path had the same length. Nonetheless, this means that each time a corner was taken, coordination was lost and the metric for the relative along-path distance did not make sense in the corner section. Therefore, the goal was to only achieve coordination on the straight-line sections of 200 [m] length depicted in Figure 3, which are sections of common length to each vehicle. The corner sections were traversed in a way that aimed to keep the formation error as small as possible before reaching the next straight-line section. This was done by normalizing each corner section such that to the LAUVs they appeared to have the same length. This normalized distance was then scaled to artificially increase path-following errors on the normalized section. This resulted in the outside LAUV speeding up when traversing the corner and the inside LAUV slowing down to reduce the distance between the LAUVs as much as possible before the next straight-line section was reached. To achieve coordination, communication of the along-path distances between the vehicles was necessary. For the purposes of this experiment, the LAUVs were at the surface and communicated using their WiFi antennas. The communication was not done directly but was routed through a Manta communication gateway set up at the dock side. The communication gateway made a local communication network for the vehicles to send their messages through. Each vehicle communicated its position to only one other vehicle and received a position from the third vehicle.

The LAUVs were given a nominal speed of *U*_*rd*_ = 1.25 [m/s] and could adjust their speed by 0.25 [m/s] either way. Since each LAUV communicated its messages to only one other vehicle and the desired formation was a line, the argument of the synchronization function *g*(·) was simply the along-path distances of each vehicle subtracted. Consequently, the velocity assignment was made by taking.

(11) $$\begin{array}{l} u_{c_{j}}\left(t\right)=1.25-\frac{2\cdot 0.25}{\pi }\text{ta}{\text{n}}^{-1}\left(x_{j}-x_{i}\right). \end{array}$$

The look-ahead distance for the vehicles was set to Δ = 4 [m] and the integral gain was set to σ = 0.5 [m/s]. To be able to communicate through the WiFi network, the vehicles needed to be at the surface. Therefore, the depth controller of the vehicles were used to keep the vehicles on the surface.

## 4. Simulation Benchmark and Experimental Results

In this section we present the results from the experimental verification. To do this, in the first subsection we present the simulation benchmark that is used to compare with and interpret our experimental results. In the second subsection we present the results of the sea-trials and we discuss the results through a comparison with the results of the simulation benchmarks of the first subsection.

### 4.1. Simulation Benchmark

**Figure 4:**
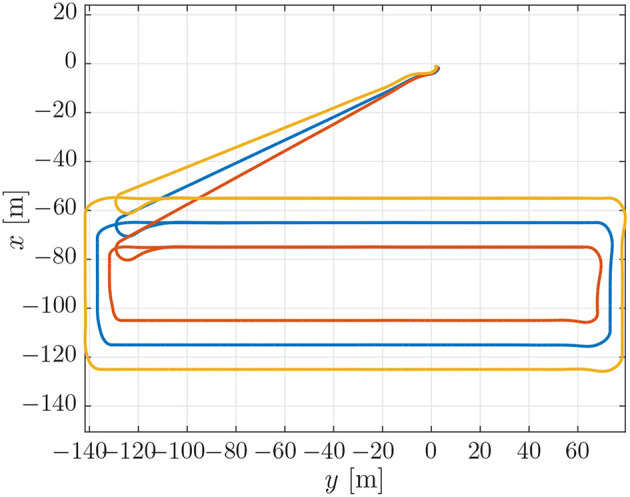
Position estimates from the vehicle observers.

**Figure 5:**
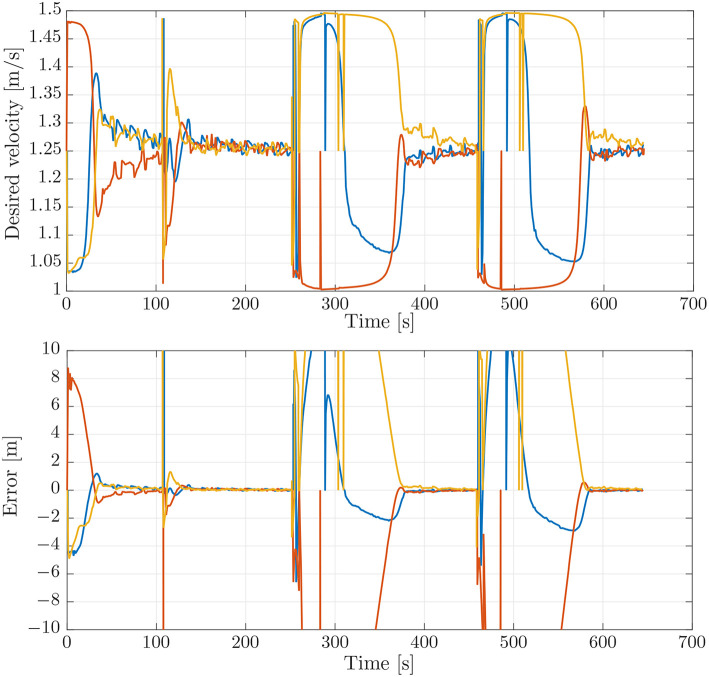
**(Top)** Desired velocity assignment for the vehicles in the simulation. The colors correspond to the vehicle colors in Figure 3. **(Bottom)** Synchronization errors between the vehicles in the simulation. The blue line is the synchronization error of the blue vehicle with measurements from the red vehicle. The red line is the synchronization error of the red vehicle with measurements from the yellow vehicle. The yellow line is the synchronization error of the yellow vehicle with measurements from the blue vehicle.

To evaluate the experimental data gathered during the mission described above, we first performed a simulation which can be used as a benchmark. This simulation was performed using DUNE, which provides high-accuracy hardware simulations of the vehicles to test code implementations. To simulate an environmental disturbance, a constant ocean current was added which had a component in the north/south and the east/west direction. The ocean current had a velocity of 0.2 [m/s] from the east direction and 0.1 [m/s] from south direction. The parameters used in the simulations were the same as the parameters which were used in the experiments, given in section 3.3, i.e., *U*_*rd*_ = 1.25 [m/s], Δ = 4 [m], and σ = 0.5 [m/s]. The motion of the vehicles in the simulations can be seen in Figure 4, from which we see that the paths accurately resemble the pattern suggested in Figure 3. The desired velocity assignments can be seen in the top plot of Figure 5, from which it can be seen that the desired velocity assignment was as expected. Especially during the corner sections it can be seen that the red vehicle was made to wait for the other vehicles by lowering its speed, and we see the velocity assignment of the blue vehicle change once it synchronizes with the red vehicle. On the straight-line sections the velocity does not converge precisely to the nominal value but, *u*_*c*_, oscillates around it. These same oscillations can be seen in the plot for the synchronization errors given in the bottom plot of Figure 5 where we see that the error does not go to zero but oscillates around it. This can be expected for the hardware simulations presented here, which have discrete communication. This causes the vehicles to overshoot their desired positions to achieve synchronization. Moreover, this discretisation causes transients in the velocity controllers at every step which also prevent the vehicles from perfectly tracking their desired inputs, which is something that is guaranteed in the theory by the feedback-linearizing controllers, and which is seen in the ideal numerical simulations in Belleter and Pettersen (2014) by the feedback-linearizing controllers.

### 4.2. Experimental Results

**Figure 6:**
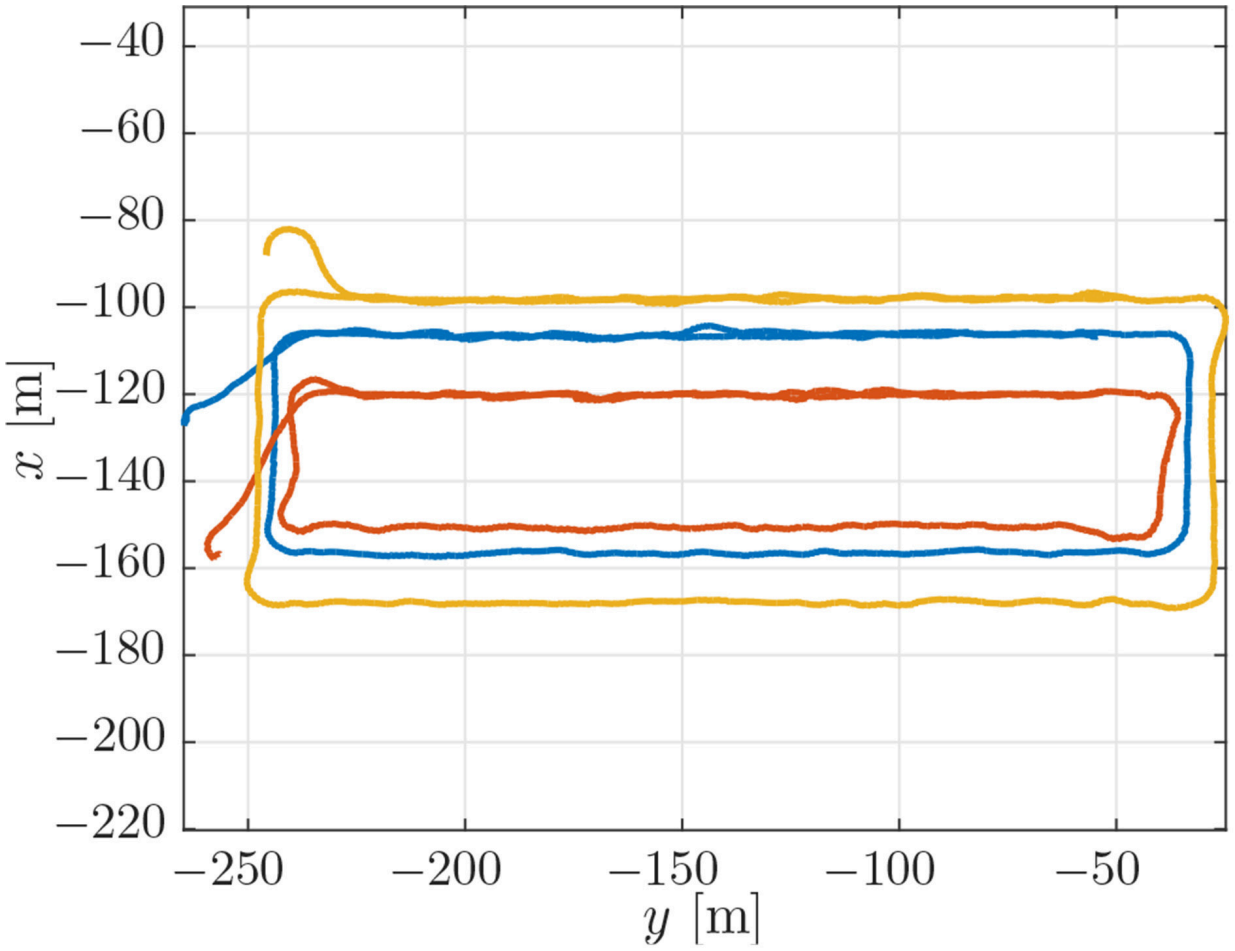
On board estimates of the paths of the vehicles in the experiments.

**Figure 7:**
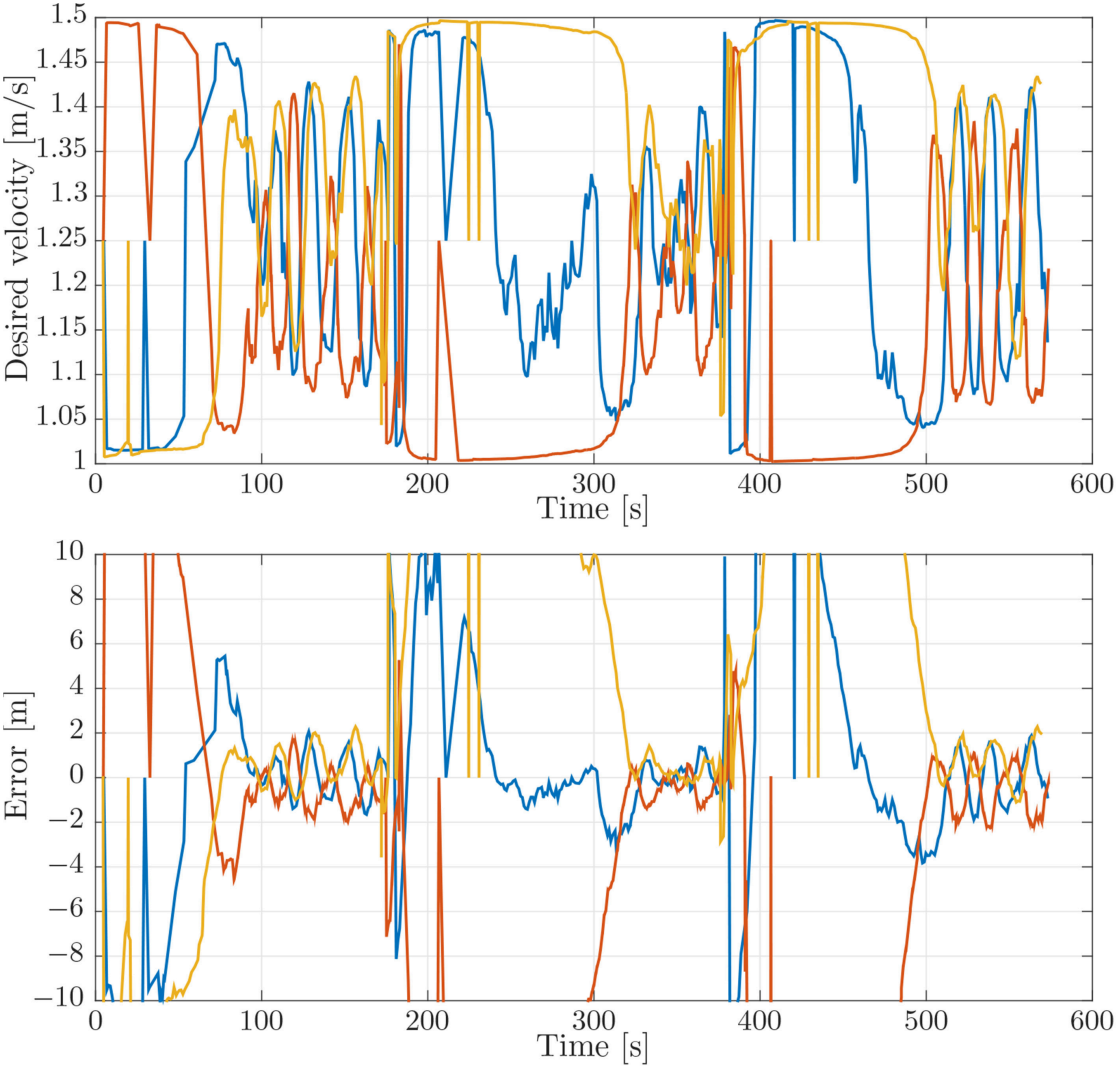
**(Top)** Desired velocity assignment for the vehicles in the experiment. The colors correspond to the vehicle colors in Figure 3. **(Bottom)** Synchronization errors between the vehicles in the experiment. The blue line is the synchronization error of the blue vehicle with measurements from the red vehicle. The red line is the synchronization error of the red vehicle with measurements from the yellow vehicle. The yellow line is the synchronization error of the yellow vehicle with measurements from the blue vehicle.

The trajectories for the vehicles performing the mission described in section 3.3 can be seen in Figure 6. From Figure 6 it can be seen that the vehicles converged to the prescribed patterns. However, the trajectories were less smooth in open water than in simulation. This can be seen by comparing Figure 4 and Figure 6. This is a performance degradation that is to be expected by the added uncertainties in the experiments, especially at the surface where the vehicles are also exposed to waves. Note that in Figure 6, the distances between the rectangles may not be accurately depicted, since the placement of the rectangle depends on the global estimate of each vehicle's position when the vehicle is initialized, the position is then interpolated from that position. However, the overall shape of the path indicates that the geometric task of path following was achieved satisfactorily for the circumstances under consideration. The desired velocity assignment can be seen in the top plot of Figure 7. It can be seen that the pattern of the relative velocity assignments resembles those of the simulation results in Figure 5. However, like for the motion patterns, the added uncertainty leads to larger oscillations during the straight-line sections and that the signal is generally less smooth. Despite the oscillations it can be seen that the velocity assignment was performed as desired by making the vehicles that are ahead wait while the vehicles that are behind speed up until a steady-state is reached. The same difference with respect to the simulations can be seen from the plots of the synchronization errors, which are given in the bottom plot of Figure 7. The synchronization errors in Figure 7 show larger oscillations on the straight-line sections and are less smooth in general than the synchronization errors from the simulations in Figure 5. From Figure 7 it can be seen that after a transient period in which the vehicles converged to the desired paths and got in formation, they reached a steady-state which was maintained until they arrived at the next waypoint. The steady-state had oscillations of the synchronization errors with an amplitude of up to 2 [m]. However, it should be noted that the oscillations were much smaller on the second straight-line section. This suggests that the difference in environmental circumstances between the two sides of the rectangle might play an important roll in the amplitude of the oscillations. The amplitude of the oscillations should preferably be lowered in future implementations, and in the remainder of this subsection we will discuss measures to achieve this. Before going into these measures, we note the fact that the communication only attained a low bandwidth of about 1 [Hz]. Consequently, the desired velocity could only be updated every second, something which caused vehicles to overshoot the desired formation. Although a similar bandwidth was utilized in the simulations of section 4.1, the bandwidth was attained uniformly over the whole path in the simulations, while in the experiments communication was more intermittent and the attained bandwidth differed between the vehicles. Although the vehicles run internal processes at a frequency of 10 [Hz], only a low bandwidth was attained due to the limitations of the WiFi signal over a long range. Moreover, since the Manta gateway served as a communication hub it had to process and relay all the data between the vehicles. In future experiments, direct communication between the vehicles or a more local communication could be pursued to increase the communication bandwidth.

**Figure 8:**
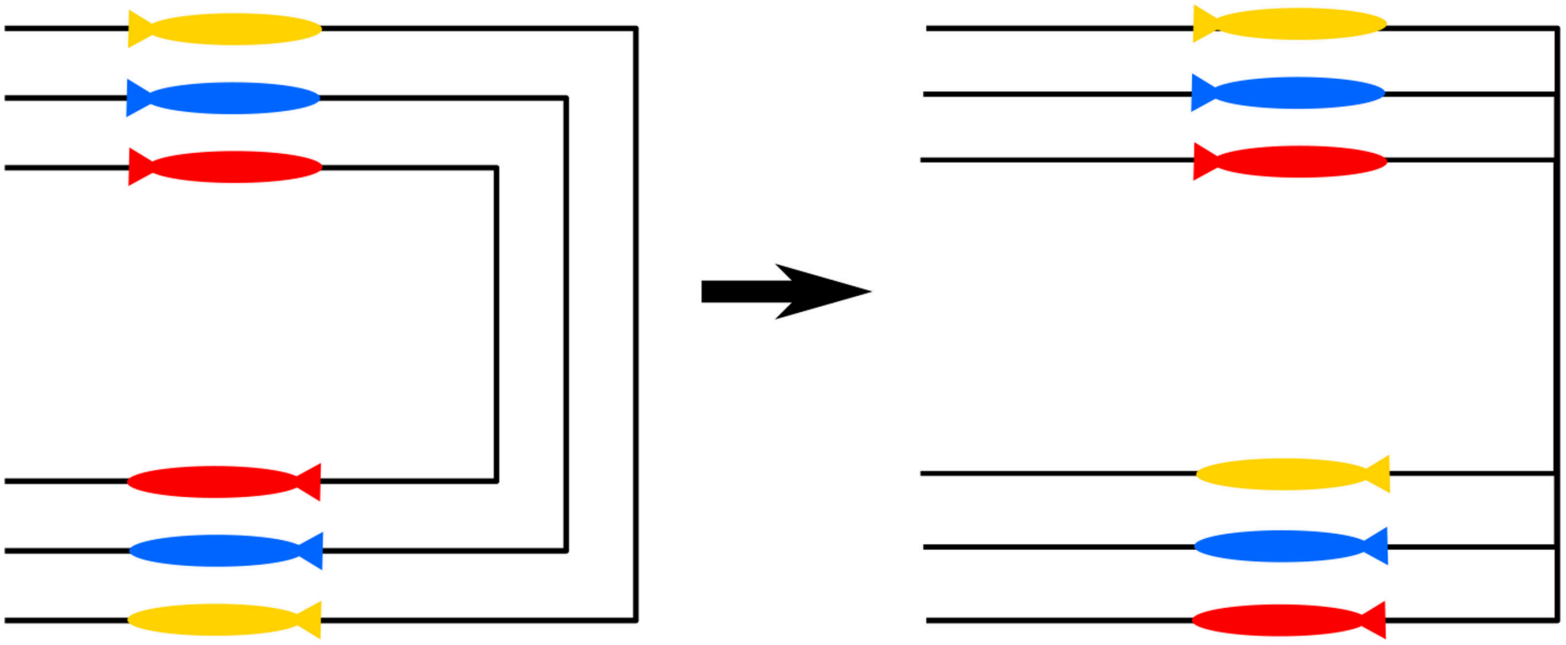
Current implementation of cornering **(Left)** and suggested alternative implementation **(Right)**.

The oscillations on the straight-line sections can be attributed to several factors. The most important is the low bandwidth of communication which causes delays in the changes of the desired velocity that are required to achieve the formation. Moreover, a transient in the velocity controller is induced every time the velocity is changed. These changes in the velocity are much less smooth in this case than it would be if the communication bandwidth was higher. This was already the case for the hardware simulations in section 4.1 and was exacerbated by the more intermittent communication in the experiments. Added to that, there was more delay in the communication, which required interpolation to compare incoming along-path distance measurements to stored along-path distances of the vehicle itself, such that the timestamps of those measurements matched. Consequently, the calculated errors became less accurate due to the interpolation, and the control action was applied with a delay since the error was “old” at the time when the control action was computed and applied. Besides increasing the bandwidth, over which direct control might not be available, several other measures can be taken. One measure could be to change the synchronization function. More specifically, the slope of the arctangent around the origin can be decreased. This has the effect that changes in the velocity will be smoother, and if the vehicles overshoot the desired position, then the resulting change in velocity is less severe. This might be combined with an increase of the look-ahead distances, which will make the guidance of the vehicles less reactive to changes in the velocity and the transients in the controller. The negative effect of changing the slope of the arctangent is that it will take longer for the vehicles to converge to the formation. Also, an increase of the look-ahead distance will reduce the convergence rate. Therefore, the choice will be a trade-off between convergence speed and desired steady-state behavior. For the case considered here, speed of convergence plays an important role due to the transients introduced in each corner. For future implementations, this requirement can be removed by letting the vehicles have the same corners and interweaving trajectories as suggested in Figure 8. The implementation in Figure 8 makes the cornering distance identical for each vehicle and should maintain formation when coming to the next straight-line section. Therefore, the necessity of fast transients is removed. Another measure to remove the oscillations is a change in the communication topology. In the implementation presented here the graph is cyclic. This has the advantage that all the vehicles wait for vehicles that are left behind. This assures again that the steady-state is reached faster. However, it also implies that all the vehicles should synchronize at the same time. More specifically, partial synchronization between two of the vehicles is disturbed when one of the vehicles is waiting for the third while the other vehicle desires to maintain the nominal velocity. This can be solved by implementing the communication graph in a leader-follower like structure where two of the vehicles synchronize only to the leader, which allows for synchronization between two of the vehicles while the third vehicle still converges. Another option could be that each vehicle sends its information to both of the others. However, this is more complicated from an implementation perspective since interpolation will have to be applied to match all the time-stamps of incoming messages, which will also introduce some additional errors. Another plausible explanation for the deviation, the disturbance of the waves that could clearly be observed during the experiments, could not be verified in simulation. Nonetheless, this is a likely cause of additional errors since the disturbance of the waves causes a necessity for more control action, which interferes with both the path following and coordination. Despite the difficulties mentioned here, the experimental results illustrate the effectiveness of the proposed coordinated path-following strategy.

## 5. Discussion

To test some of the suggested measures and highlight the advantages and drawbacks of them, we present three further simulation cases. The three cases under investigation are the following.

- One test with a smaller look-ahead distance Δ = 2[m] to show the adverse effects of a smaller Δ.
- One test with a larger look-ahead distance Δ = 6[m] to show the stabilizing effect of a larger Δ.
- One test with a lower communication frequency of 0.25[Hz] to show the adverse effects of slower communication.

### 5.1. Case 1: Smaller Look-Ahead Distance

**Figure 9:**
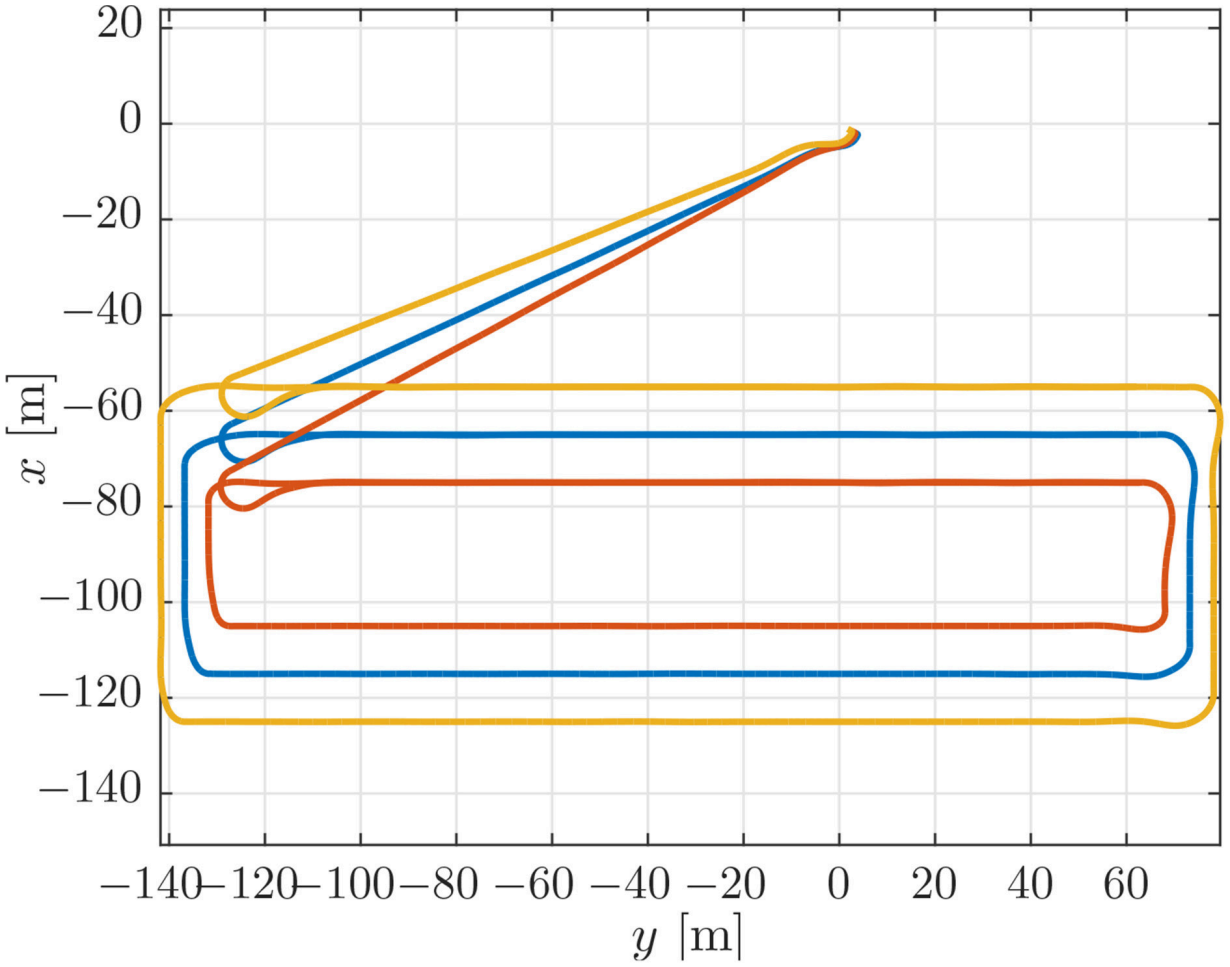
On-board estimates of the paths of the vehicles.

This case is designed to illustrate the effect of a small look-ahead distance. This can cause oscillations around the path that make coordination more difficult, cf. the stability analysis in Børhaug et al. (2008). To illustrate this, we have repeated the simulation benchmark of section 4.1 with the look-ahead distance of Δ reduced from 4 [m] to 2 [m]. The paths for this simulation can be seen in Figure 9. From the paths in Figure 9 it can be seen that the paths are much more oscillatory when compared to those of the simulation benchmark in Figure 4.

**Figure 10:**
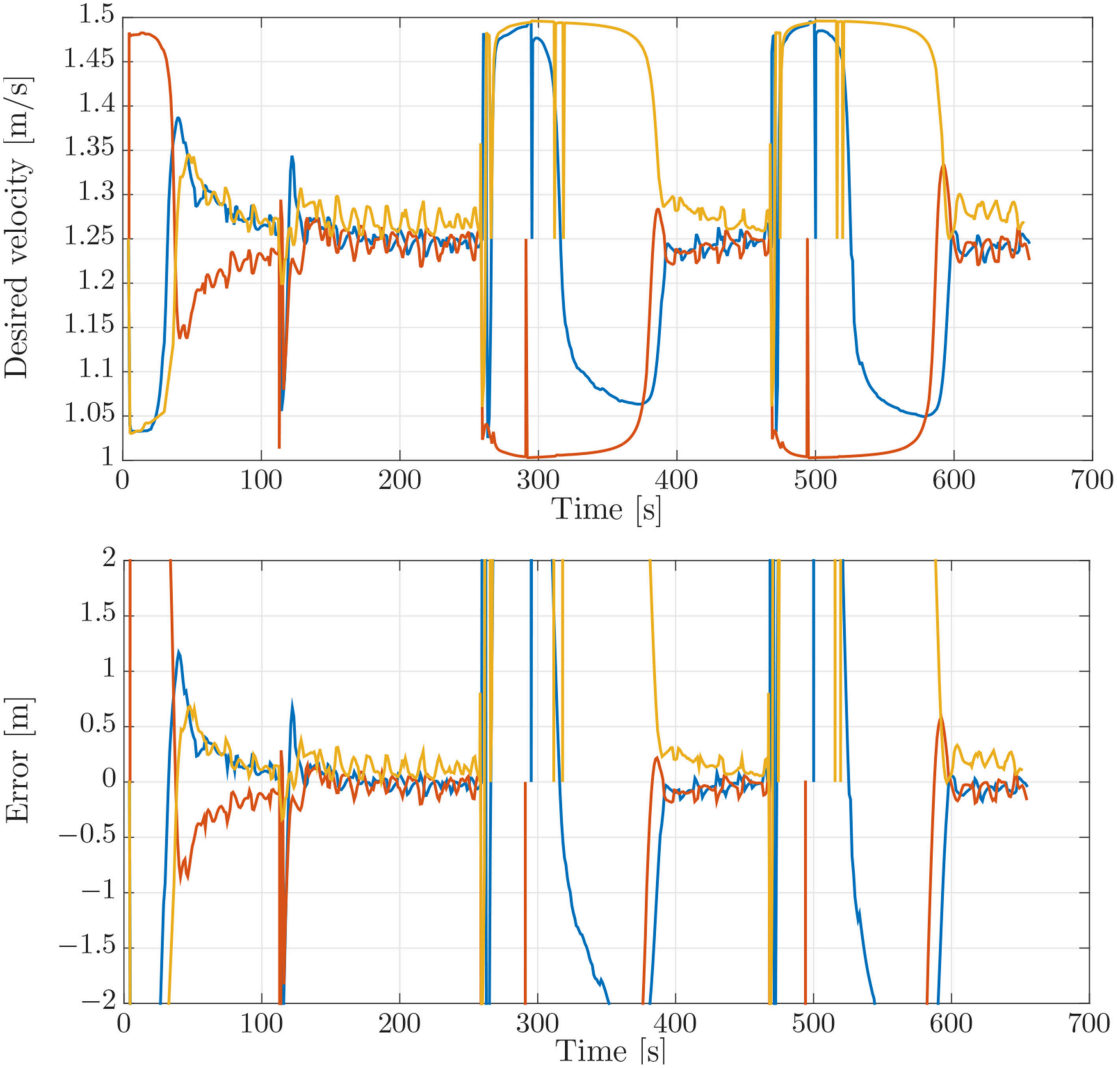
**(Top)** Desired velocity assignment for the vehicles in the simulation. The colors correspond to the vehicle colors in Figure 3. **(Bottom)** Synchronization errors between the vehicles in the simulation. The blue line is the synchronization error of the blue vehicle with measurements from the red vehicle. The red line is the synchronization error of the red vehicle with measurements from the yellow vehicle. The yellow line is the synchronization error of the yellow vehicle with measurements from the blue vehicle.

The effects of the larger oscillations around the path can be seen when we look at the desired velocity assignment and the synchronization error in Figure 10. These exhibit larger oscillations and deviations than the ones in the simulation benchmark, Figure 5. The largest oscillations can be found for the LAUV following the outer trajectory, since this vehicle makes the biggest corner. This can also be seen when looking at the desired velocity in Figure 10 which exhibits larger oscillations for the vehicle on the outer trajectory and in fact causes the vehicle on the outer trajectory not to converge to the formation in this test.

### 5.2. Case 2: Larger Look-Ahead Distance

**Figure 11:**
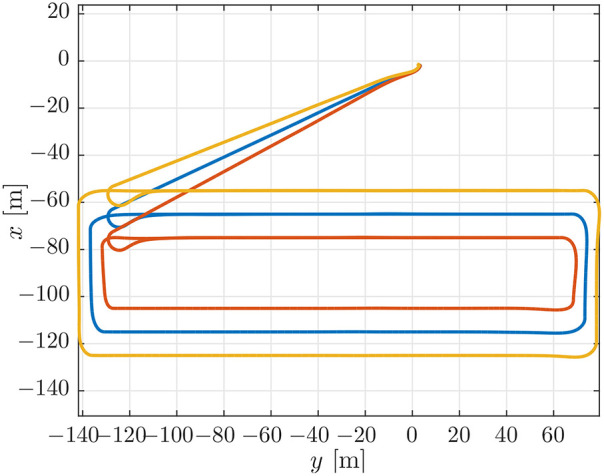
On-board estimates of the paths of the vehicles.

In this case we do the opposite of the previous case and we increase the look-ahead distance to 6 [m] to stabilize the path-following behavior. More specifically, the analysis in Børhaug et al. (2008) shows that if the look-ahead distance is increased, the trajectory is much smoother and the steering will be less reactive, making all trajectories smoother. This can be seen in Figure 11 which shows very smooth trajectories and also less overshoot in the corners.

**Figure 12:**
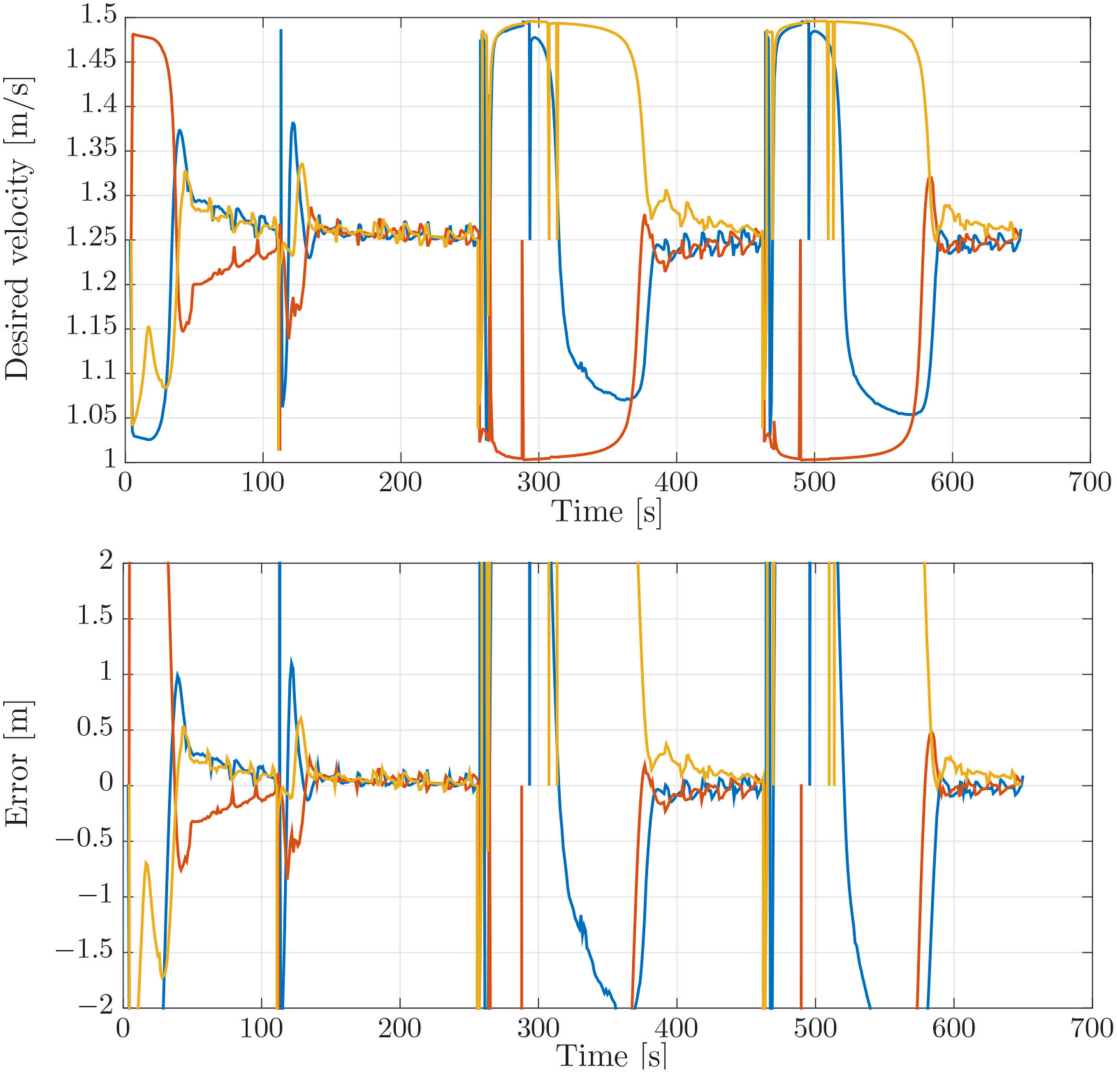
**(Top)** Desired velocity assignment for the vehicles in the simulation. The colors correspond to the vehicle colors in Figure 3. **(Bottom)** Synchronization errors between the vehicles in the simulation. The blue line is the synchronization error of the blue vehicle with measurements from the red vehicle. The red line is the synchronization error of the red vehicle with measurements from the yellow vehicle. The yellow line is the synchronization error of the yellow vehicle with measurements from the blue vehicle.

This also translates to the desired velocity plot and the synchronization error plot in Figure 12. We see that when compared to Figure 10, the oscillations and final errors are much smaller in Figure 12. Also when comparing Figure 12 with Figure 5 we see that the trajectories for the desired velocity, although converging a bit slower, are a bit more smooth.

### 5.3. Case 3: Lower Communication Frequency

In this final case we show the adverse effects of a lower communication bandwidth. The communication bandwidth is lowered from around 1 [Hz] to maximally 0.25 [Hz]. This makes synchronization between the vehicles more difficult since the vehicles are more likely to overshoot the formation when trying to converge.

**Figure 13:**
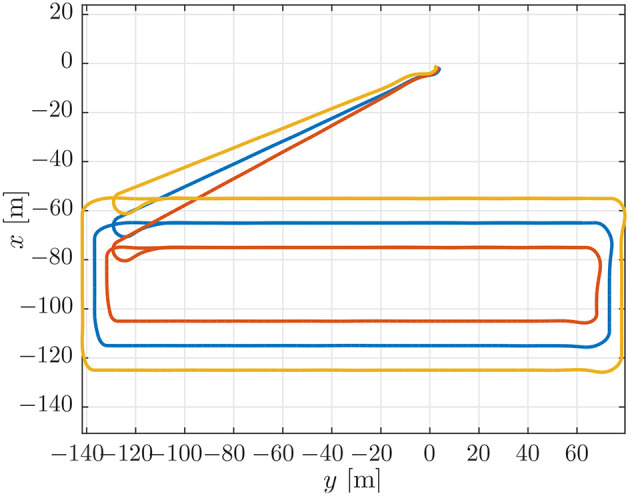
Estimates of the vehicle paths from the vehicle observers.

**Figure 14:**
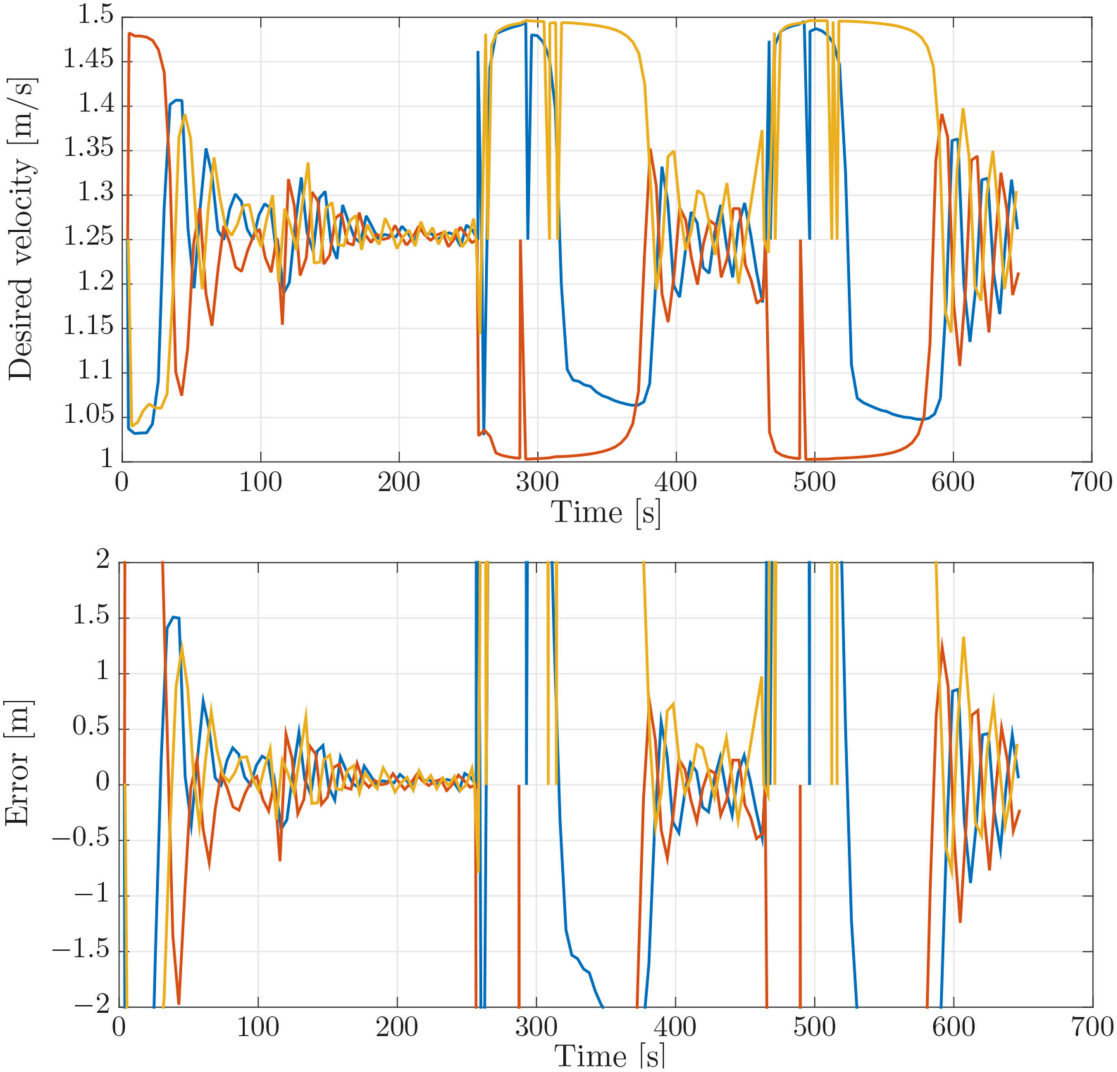
**(Top)** Desired velocity assignment for the vehicles in the simulation. The colors correspond to the vehicle colors in Figure 3. **(Bottom)** Synchronization errors between the vehicles in the simulation. The blue line is the synchronization error of the blue vehicle with measurements from the red vehicle. The red line is the synchronization error of the red vehicle with measurements from the yellow vehicle. The yellow line is the synchronization error of the yellow vehicle with measurements from the blue vehicle.

The trajectories are shown in Figure 13 and are very similar to the trajectories of the simulation benchmark in Figure 4. The desired velocity trajectories, however, are very different. The oscillations around the desired final velocity are much larger and more similar to the profiles from the experimental verification in Figure 7. The same holds for the coordination errors given in Figure 14 which look more similar to the errors from the experimental verification in Figure 7 than the errors from the simulation benchmark given in Figure 5. This indicates that a low communication bandwidth plays a major role in these problems. Another issue is the performance of the controllers which take time to converge, adding further delays and deviations from the desired input and thereby increase the errors.

## 6. Conclusion

In this work we presented and experimentally verified the combination of integral line-of-sight path following with a coordination law to achieve coordinated path following. First, we presented the underlying theory for path following in the presence of unknown ocean currents and the theory for coordination of vehicles along the path. The guidance law and coordination law were then implemented in the vehicle's software and simulations were performed to test the feasibility and create a benchmark for the experimental results.

Experimental results were obtained from sea-trials in the harbor of Porto. In these experiments three vehicles were required to coordinate their motion along parallel straight-line paths to reach a line formation. The results were compared to the simulation benchmark and found to have larger errors than the simulation benchmark. A discussion of the differences, possible causes and mitigations were presented. It was hypothesized that these differences could be attributed to a look-ahead distance that is too low and a communication bandwidth that is too low. To test this hypothesis several more simulations were performed to show the effects of the look-ahead distance and communication frequencies on the system behavior. Using these simulation results we were able to give plausible explanations for the deviations from the first simulation benchmark.

Further research can be done at both a theoretical and a practical level. Further theoretical developments could be to investigate a similar approach to curved path following and to include a more complex model of the disturbances. Further practical developments could include considering this application under water with acoustic communication, or with an improved communication bandwidth at the surface to see if the synchronization error can be reduced.

## Author Contributions

DB has collaborated with KP in developing the theory that is experimentally verified. He was responsible for writing the application code to test the coordinated path following strategy on the vehicle. He assisted in planning and execution of the sea-trials. He was responsible for performing the simulations, analysis of the experimental data and analysis of the data from the simulations. He was also responsible for writing the manuscript. JB assisted DB in writing the application code. He was responsible for integration of the application code within the vehicle code and preparing of the hardware for the mission. He planned the execution of the mission and operated the vehicles for the mission. He Exported and extracted the mission data from the vehicles. KP has collaborated with DB in developing the theory that is experimentally verified. She Assisted in analysing the data and in proof reading and correcting the manuscript.

### Conflict of Interest Statement

JB was employed by company OceanScan Marine Systems and Technology, Lda. The remaining authors declare that the research was conducted in the absence of any commercial or financial relationships that could be construed as a potential conflict of interest.
